# The art of selecting the ECG input in neural networks to classify heart diseases: a dual focus on maximizing information and reducing redundancy

**DOI:** 10.3389/fphys.2024.1452829

**Published:** 2024-10-07

**Authors:** Elisa Ramirez, Samuel Ruiperez-Campillo, Ruben Casado-Arroyo, José Luis Merino, Julia E. Vogt, Francisco Castells, José Millet

**Affiliations:** ^1^ ITACA Institute, Universitat Politècnica de València, Valencia, Spain; ^2^ Department of Computer Science, ETH Zürich, Zurich, Switzerland; ^3^ Hôpital Erasme, Université libre de Bruxelles, Brussels, Belgium; ^4^ Servicio de Cardiología, Hospital Universitario La Paz, Madrid, Spain

**Keywords:** cardiovascular diseases, electrocardiogram, deep learning, redundancy reduction, model performance, cardiac signal processing

## Abstract

**Background and Objectives:**

Accurate diagnosis of cardiovascular diseases often relies on the electrocardiogram (ECG). Since the cardiac vector is located within a three-dimensional space and the standard ECG comprises 12 projections or leads derived from it, redundant information is inherently present. This study aims to quantify this redundancy and its impact on classification tasks using Convolutional Neural Networks (CNNs) in cardiovascular diseases.

**Methods:**

We employed signal theory and mutual information to introduce a novel redundancy metric and explored techniques for redundancy augmentation and reduction. This involved lead selection and transformation to evaluate the effects on neural network performance.

**Results:**

Our findings indicate that optimizing input configurations through redundancy reduction techniques can enhance the performance of deep learning models in cardiovascular diagnostics, provided that the information is preserved and minimally distorted.

**Conclusion:**

For the first time, this research has quantified the redundancy present in the input by validating various redundancy reduction techniques using a CNN. This discovery paves the way for advancing biomedical signal processing research, simplifying model complexity, and enhancing diagnostic performance in cardiovascular medicine within reduced lead systems, such as Holter monitors or wearables.

## 1 Introduction

Cardiovascular diseases (CVDs), including both arrhythmias and non-arrhythmic cardiac conditions such as myocardial infarction or cardiomyopathies, pose significant health challenges (Goldsborough et al., 2023), demanding precise and timely diagnostic methods for effective intervention and risk reduction. With the rising prevalence of cardiovascular diseases as life expectancy increases, accurate early diagnostic tools become essential (Flora and Nayak, 2019). Among these, the ECG remains the most common tool to assess cardiac health (Holst et al., 1999). Widely available across healthcare centers, even in modest settings, and highly cost-effective, the ECG serves as a fundamental non-invasive screening technique for initial cardiovascular diagnostics, offering insights into the heart’s electrical activity (Berkaya et al., 2018).

The standard ECG recording comprises 12 leads capturing information from the cardiac dipole through 12 non-orthogonal projections, including 6 limb leads (I, II, III, aVR, aVL, and aVF) and 6 precordial leads (V1-V6) irregularly spaced in the axial plane. Other common configurations consist of only a few leads, including out-of-hospital ECG monitoring with devices such as Holter monitors (Moody and Mark, 2001; Sharma and Baranchuk, 2021) or wearable devices (Dagher et al., 2020; Smital et al., 2020; Zepeda-Echavarria et al., 2023). Less common modalities with a higher number of leads are also available, such as Body Surface Potential Mapping, which uses varying numbers of leads, including the 67 leads used in Giffard-Roisin et al. (2017).

The integration of automated diagnostic approaches like Machine Learning and particularly Deep Learning (DL), applied to massive and publicly available ECG datasets, has led to breakthroughs in the automated diagnosis of cardiac conditions (Mincholé and Rodriguez, 2019; Siontis et al., 2021). Within DL, Convolutional Neural Networks (CNNs) are commonly employed, leveraging raw signal data as input. While numerous studies have adopted a broad multipathology approach (Anand et al., 2022; Zhang et al., 2021; Wang et al., 2020), others have focused on specific conditions such as atrial fibrillation (Baek et al., 2021; Raghunath et al., 2021) or classification based on acuteness (van de Leur et al., 2020). Additionally, the ECG is commonly used in multimodal settings, such as in the prediction of malignant ventricular arrhythmias (Sahoo et al., 2020). These ECG recordings are typically digitized, although a significant volume of data still exist in paper format. To utilize these records in CNN models, prior digitization and synchronization are required (Ramírez et al., 2024).

Although the visual representation of the standard ECG, comprising 12 non-orthogonal projections, is undoubtedly suitable for human interpretation, the high redundancy of this data format, if directly put into DL models, may introduce biases in the learning process or lead to overfitting. Notably, only two out of the six limb leads are mathematically independent. Previous studies have focused on lead reduction for diagnosing cardiac pathologies (Jiménez-Serrano et al., 2022; Bruoth et al., 2021; Donnelly et al., 2008; Natarajan et al., 2021; Nejedly et al., 2021), yet concerns regarding redundancy emerged with research such as (van de Leur et al., 2020), who trained a deep neural network using eight independent ECG leads, including two limb and six precordial leads. Furthermore, the study by Lai et al. (2021) underscored the impact of ECG redundancy on the generalizability of DL models, despite the primary aim of assessing prediction with a limited data volume.

Whether redundancy affects CNN training and how this impacts accurate diagnosis across pathologies has hitherto not been studied in-depth. To address this research gap, we propose a novel framework to quantify ECG redundancy and evaluate its impact on 2D-CNN learning. Different strategies have been defined for reducing redundancy, including lead elimination and linear transformation to achieve maximum orthogonality. Additionally, we performed a comparative analysis of various levels of redundancy and their impact on CNN performance. This provides insights into optimal input configurations for DL models in cardiovascular diagnosis, potentially advancing diagnostic accuracy, particularly for automated diagnosis. In applications where a reduced number of leads is required, such as in Holter monitors or wearables, the results of this study may guide the selection of the ECG data format to be input into the learning model.

## 2 Materials

For this study, we used the PTB-XL dataset (Wagner et al., 2020), sourced from Physionet (Goldberger et al., 2000). This dataset consists of 21,837 12-lead synchronous ECG recordings from 18,885 patients, each spanning 10 s. The dataset maintains a gender balance (52% male, 48% female) and covers a wide age range (from 1 to 95 years). It consists of a control group of healthy (normal) ECGs (NORM), and various heart pathologies annotated by two cardiologists including myocardial infarction (MI), ST/T changes (STTC), conduction disturbance (CD), and hypertrophy (HYP). The ECGs are sampled at both 500 Hz and 100 Hz.

The dataset is partitioned into 10 folds, ensuring that a subject’s data is present in only one fold to prevent data leakage among folds. For our experimental settings, the initial 9 folds (88%) were used for training and validation (88% and 12% respectively), while the 10th fold served as a hold-out test group (12%).

## 3 Methods

**FIGURE 1:**
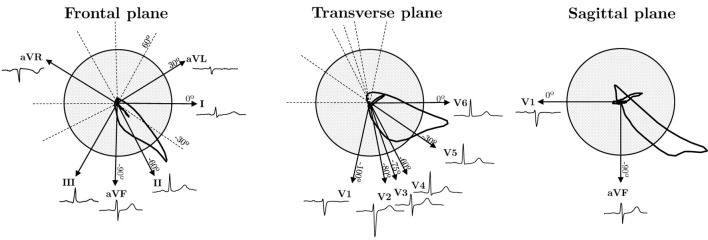
Cardiac dipole projections of the standard 12-lead ECG system on the three anatomical planes: Frontal, Transverse and Sagittal.

**FIGURE 2:**
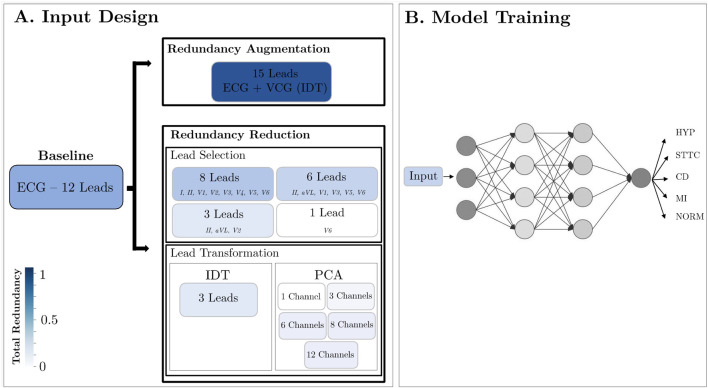
Methodology Design. **(A)** Modification of the ECG data format: lead augmentation, lead selection and linear transformation; **(B)** All devised inputs underwent testing within a CNN framework, aimed at classifying data across five distinct diseases.

The projections of the standard 12-lead ECG on the Frontal, Transverse and Sagittal planes are depicted in Figure 1. In our framework, we explore the influence of redundancy on CNN performance, as shown in Figure 2. To evaluate it, we considered modifications of the ECG data including augmentation, reduced lead subsets and linear transformations.

### 3.1 Redundancy quantification

Several metrics are proposed to quantify redundancy for each input, primarily based on the concept of mutual information ( I ) (Equation 1) which quantifies the dependence between two random variables X and Y from entropy ( H ) measurements:

$$I\left(X;Y\right)=H\left(X\right)+H\left(Y\right)-H\left(X,Y\right)$$ (1)

The H (Equation 2) of a random variable X presents the following equation:

$$H\left(X\right)=-\overset{n}{\underset{i=1}{\sum }}p\left(x_{i}\right)\log p\left(x_{i}\right)$$ (2)

In Equation 2, $p\left(x_{i}\right)$ corresponds to the probability of each possible value $x_{i}$ .

Given that the ECG values are continuous, binning was required to calculate the probabilities *p*(*x*_*i*_). After an empirical search, we selected a bin size of 0.5 mV. Sampling frequency can have an impact on the entropy computation as the higher it is, more variations can be captured. The difference in entropy between 100 Hz and 500 Hz is negligible as it differs in the order of the hundreds; however, we employed a sampling frequency of 500 Hz for all calculations.

Additionally, the Normalized Mutual Information (NMI) (Equation 3), ranging between 0 and 1, allows a direct comparison of I .

$$NMI\left(X;Y\right)=\frac{I\left(X;Y\right)}{H\left(X,Y\right)}$$ (3)

According to this definition, a value of 0 represents variables with non-dependence, while a value of 1 denotes identical variables.

**FIGURE 3:**
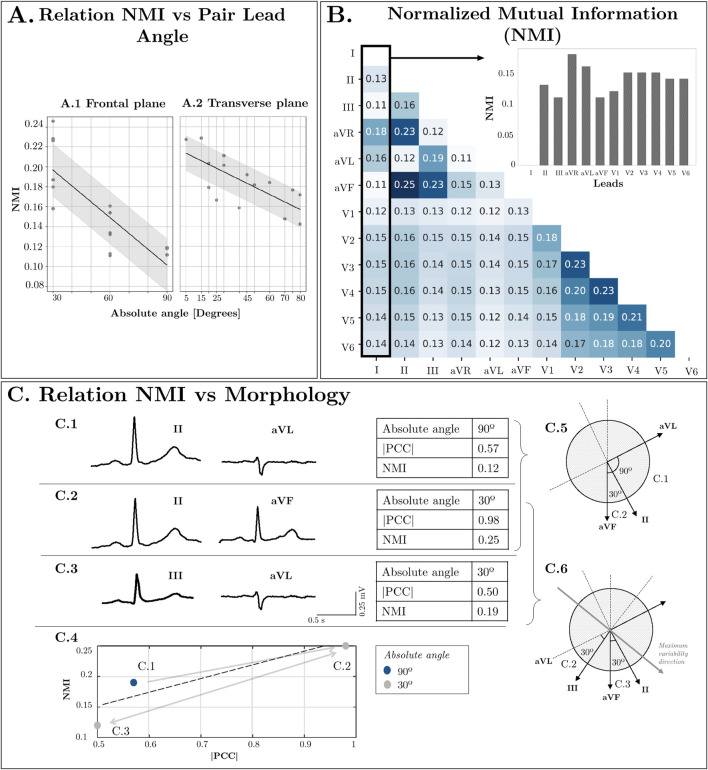
**(A)** Relation NMI vs. Lead direction in Frontal Plane and Transverse Plane angles. **(B)** Normalized Mutual Information between Leads. **(C)** Relation NMI vs. Morphology: Comparison between two leads with low orthogonality (II-aVF), two orthogonal leads (II-aVL) and low orthogonality between them but high orthogonality to the maximum variability direction (III-aVL).

Figure 3A represents the NMI dependence on the angle difference between the projections of two leads in the Frontal and Transverse planes. The absolute angle, which indicates the smallest angle in absolute value between two lead directions (see angle directions in Figure 1), ranges from 0 to 90°. Higher orthogonality leads to reduced dependence, resulting in a lower NMI value. Conversely, when lead directions exhibit greater similarity, the information conveyed by both leads becomes more similar, leading to a higher NMI value. Furthermore, Figure 3B displays the NMI matrix for each ECG lead, illustrating the dependency between each pair of leads. This calculation was performed using healthy signals from the dataset to mitigate potential inaccuracies stemming from variations in cardiac dipole morphology due to different pathologies. Additionally, Figure 3C relates angle with NMI and changes in ECG morphology quantified by means of Pearson Correlation Coefficient (PCC). In Figures 3 C.1, C.2, when using a common lead in both cases (lead II), it is observed that as the angle increases, the morphology becomes more distinct. However, as illustrated in Figures 3 C.2, C.3, when the angle is held constant at 30° but the orientation varies, the morphology remains similar in the first case, where the directionality closely aligns with the maximum variability. In contrast, the similarity decreases in the second case, where the directionality diverges.

Figure 3 C.4 also intends to show that there is a relationship between the NMI and the morphology similarity between leads even though in the NMI, each sample is treated as an independent and identically distributed (iid) sample without taking into account the temporal relationship between them. That is, higher values of NMI show also absolute value of PCC and vice-versa.

While Figure 1 illustrates the ideal projection directions of each lead, angles deviate slightly due to variations in electrode placement in real-world settings. Additionally, precordial leads not only display variance in angle in the axial plane but also exhibit minor deviations in the frontal plane, causing actual angles to be larger than those depicted. These factors contribute to the observed standard deviation (approximately 0.03) in Figure 3A, highlighting NMI as a superior and more reliable criterion for assessing lead dependence compared to angle differences. Furthermore, even if two pairs of leads present the same angle (see Figures 3 .C1, 3.C2), the mutual information varies depending on their relationship with the direction of maximum variability of the cardiac vector. For instance, two leads with an angle closer to the direction of variability (such as II and aVF) will exhibit higher NMI than two leads more orthogonal to it (such as III and aVL). For these reasons, a similarity criterion based solely on the angle between leads is insufficient to quantify the shared information.

To estimate the extent of information from one lead $\left(X_{i}\right)$ that is present in the other n leads and, conversely, the extent of information from the other leads that is contained in one specific lead, we define the redundancy (R) (Equation 4) for a single channel as:

$$R\left(X_{i}\right)=\frac{H\left(X_{i}\right)-H\left(X_{i}|X_{1},\ldots ,X_{i-1},X_{i+1}\ldots ,X_{n}\right)}{H\left(X_{i}\right)}$$ (4)

Building upon this concept, we measure the amount of redundant information present in a set of leads, which indicates how much of the total information is shared among at least two leads. To achieve this, the definition of R can be extended for a set of channels according to the following Equation 5:

$$\begin{array}{l} R\left(X_{1},X_{2},\ldots ,X_{n}\right)\\ \;=\frac{H\left(X_{1},X_{2},\ldots ,X_{n}\right)-H\left(X_{1}|X_{2},\ldots ,X_{n}\right)-\cdots -H\left(X_{n}|X_{1},\ldots ,X_{n-1}\right)}{H\left(X_{1},X_{2},\ldots ,X_{n}\right)} \end{array}$$ (5)

The presented metrics range from 0 to 1; however, can be rescaled from 0 to 100 in order to express a percentage.

For the computation of all metrics detailed above avoiding the influence of other sources of variability caused by certain pathologies we selected only healthy patients, as their ECG’s morphology is more homogeneous. This homogeneity among subjects allowed us to assume that each lead for all records captures equivalent information. Concatenating all the records increases the number of samples for the computation of the metrics, which increases the reliability of the probability distribution in the calculation of the entropies. These metrics were calculated across all selected patients by concatenating their ECGs. This approach improves histogram resolution, leading to greater accuracy in the calculations.

### 3.2 Redundancy reduction

In this section we describe two redundancy reduction strategies. On the one hand, a strategy based on lead selection, which extracts the optimal subset of leads for the desired number of channels. In this method, the selected signals match the waveforms from the original data. On the other hand, linear transformation methods, which derive a number of signals, each of them being a linear combination of the ECG leads. This way, none of the signals match any lead from the original data. In any case, the standard 12-lead ECG served as the baseline for performance assessment.

#### 3.2.1 Redundancy reduction through Lead selection

To reduce input redundancy while preserving essential information, the input was streamlined by retaining leads with higher score values. Consequently, selections were made to retain as few leads as feasible while minimizing redundancy. We derived and evaluated subsets comprising eight, six, three, and one lead, as described below.

**FIGURE 4:**
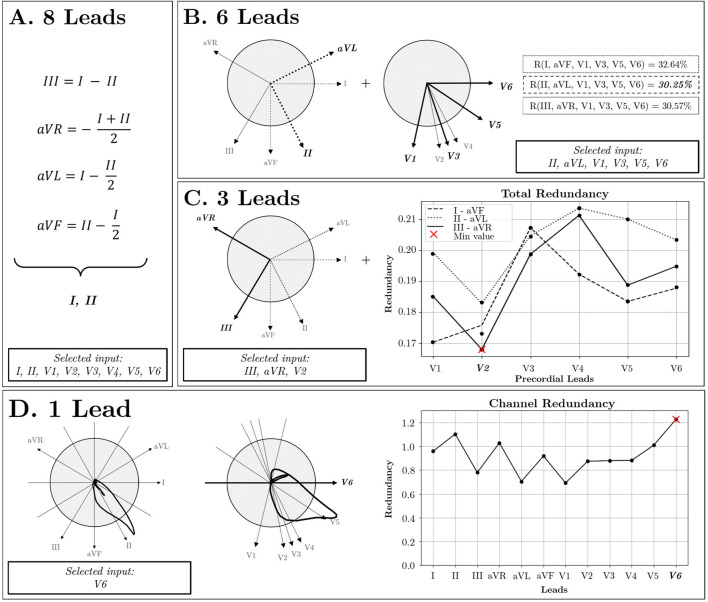
Lead Selection Strategy. **(A)** 8 Leads: Independent lead selection. **(B)** 6 Leads: In the frontal plane, three pairs of orthogonal leads are identified: I-aVF, II-aVL, and III-aVR. Additionally, in the Transverse plane, V2, V3, and V4 exhibit similar directions, allowing for their reduction to the V3 lead. By combining these orthogonal pairs with selected precordial leads, redundancy is reduced using II-aVL pair of leads (R = 30.25%). **(C)** 3 Leads: The selection process focuses on three orthogonal directions by combining the orthogonal pairs from the frontal plane with precordial leads situated in the perpendicular plane, optimizing for orthogonality. **(D)** 1 Lead: Utilizing the Channel Redundancy metric identifies the most representative lead, aligning most closely with the cardiac axis direction.

Firstly, the 8-lead set was obtained by removing leads III, aVR, aVL, and aVF, as they can be mathematically reconstructed from linear combinations of leads I and II (Figure 4A). Consequently, despite this reduction, the original information is fully preserved, resulting in no loss of information with respect to the 12-lead ECG.

For the 6-lead subset, two additional precordial leads were removed. Upon examination of the transverse plane, due to the proximity of V2, V3, and V4 (Figure 4B), high redundancy among them was inferred. Therefore, leads V2 and V4 were removed. Additionally, to achieve maximal orthogonality between leads, three pairs of orthogonal leads were identified from the frontal plane: I-aVF, II-aVL, and III-aVR, with redundancy scores of 32.64%, 30.25%, and 30.57%, respectively. Since the frontal lead pair II-aVL exhibited the lowest redundancy, the final 6-lead subset was defined by leads II, aVL, V1, V3, V5, and V6. For the 3-lead subset, the selection involved leads aVR, III, and V2 as the lead combination with maximum orthogonality and minimal redundancy (Figure 4C). Finally, the criterion to choose the single-lead input was the channel containing the highest redundancy with the rest of the leads. As illustrated in Figure 4D, lead V6 was identified as the most representative.

#### 3.2.2 Redundancy reduction through Lead transformations

In addition to direct examination of original ECG leads, several transformations were explored seeking orthogonality and minimizing redundancy. These transformations include the Inverse Dower Transform (IDT) and Principal Component Analysis (PCA).

##### 3.2.2.1 Inverse dower transformation

The vectorcardiogram (VCG) recorded using the Frank lead system (Frank, 1956) captures the cardiac dipole in three orthogonal spatial dimensions XYZ, hence preserving 3-dimensional information of the electrical activity. In the context of this study, the VCG was approximated using the IDT (Edenbrandt and Pahlm, 1988) (see Supplementary Appendix SA for a more detailed description). This transformation permits a linear conversion to a 3-channel space (refer to Supplementary Appendix Table S1).

##### 3.2.2.2 Principal component analysis

PCA is a mathematical technique used for dimensionality reduction and data compression. Given a dataset of P signals or channels (also called observations), PCA seeks a linear transformation that provides a set of Q uncorrelated signals, also called Principal Components (PC), with Q≤P , that comprises as much information as possible of the original data. For this, the first PC is obtained from the transformation that retrieves a signal with the highest variance. The subsequent PC are then obtained from a linear transformation that, being orthogonal to those of the previous PCs, retrieves the highest possible variance. Therefore, the PCA approach can be regarded as a decomposition of components that capture maximal variability or representativity of the original data. So far, PCA has been successfully applied to ECG data in clinical applications related to myocardial ischemia, atrial fibrillation or the analysis of ventricular repolarization, among others (Castells et al., 2007).

**FIGURE 5:**
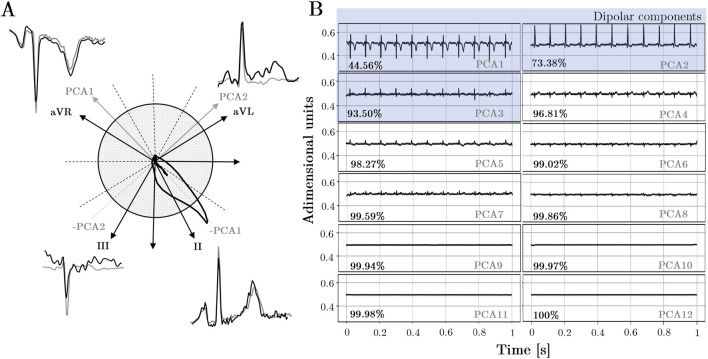
**(A)**Comparison of PCA direction projections onto the frontal plane with the morphological similarity observed in leads sharing similar directions. **(B)**PCA components: Explained variability for all records in the dataset. The first three components correspond to spatial directions known as dipolar components, while the remaining components correspond to other orthogonal directions beyond the spatial dimensions.

When PCA is applied to the 12-lead ECG, up to 8 orthogonal components can be expected, since only 8 out of the 12 original ECG leads are independent. The PCs are ordered with decreasing explainability according to variance criterion (see Figure 5). Consistent with the fact that the resultant dipole is a 3-d vector, the three first components are able to capture most of the variance of the full 12-lead ECG. On the other hand, from the fourth to the eighth components can be associated to the weaker non-dipolar components, although not for this reason they can be exempt of diagnostic value.

In light of a fair comparison with the performance of lead selection subsets, PCA subsets with the same number of components were created, i.e., PCA-12 PCA-8, PCA-6, PCA-3 and PCA-1, respectively. It is worth noting that mathematically, there are only 8 independent leads; however, a set of 12 components can be obtained due to noise in signal acquisition. Nonetheless, these additional 4 components have amplitudes close to zero and practically negligible explainability. However, it will be examined how adding null components that contribute neither redundancy nor information may impact the model’s performance.

#### 3.2.3 Redundancy augmentation

In order to evaluate how the increase in redundancy affects the input of a neural network, we designed an input composed of the 12 leads of the ECG and added three more channels corresponding to the estimated VCG with the IDT.

### 3.3 Deep learning model

A CNN was used with two objectives: 1) to evaluate how redundancy affects a neural network and 2) to validate whether the strategic reduction of redundancy retains the clinical information for each of the pathologies in this study. Therefore, for the second objective, if the performance does not decrease for a given input with respect to the 12-lead baseline, it means that it contains all the information.

Anand et al. (2022) proposed various 2D-CNN models, subsequently evaluated using the same dataset (PTB-XL database), to perform multiclass classification of the five heart conditions (HYP, STTC, MI, NORM, CD) present in the database from 10-s length, 12-lead ECG records. We tuned the architecture of their best-performing model, which consists of a 6-layer design integrating two-dimensional convolutional layers, as illustrated in Supplementary Appendix Figure S1 in Supplementary Appendix SB. The initial five layers focus on temporal aspects with kernels of dimensions (1, N ), where N represents the kernel width. Conversely, the last convolutional layer before flattening leverages spatial features by using kernels of shape ( N , 1), with N as the kernel height matching the number of input channels. This layer is designed for multi-class classification of the five heart conditions included in the selected database. The hyperparameters used were consistent with those presented in (Anand et al., 2022), although the learning rate was fine-tuned using the validation set (see fine-tuned hyperparameters in Supplementary Appendix SC).

In addition to the multiclass classification model, the effect of redundancy in binary classification will be observed. For this purpose, the same architecture has been used, changing the last layer to binary classification. For this evaluation, we have selected two experiments: 1) Healthy vs. Non-Healthy classification: in this set of experiments, all the data in the database have been used grouping all the pathologies as “Non-Healthy” category and the NORM label as “Healthy”. The data ratio in this case is 34.2% for Healthy and 65.8% for Non-Healthy. 14,620 for training and 1,652 for testing. 2) MI vs. Normal classification: Since MI is one of the most clinically relevant pathologies due to its frequency and lethality, it was decided to study the binary classification of this pathology. The total amount of data for training was 10,452 and for testing 1,169 samples. The proportion of data is 36.5% for MI and 63.5% for NORM.

Additionally, weights (w) were integrated into the loss function to address learning bias stemming from class imbalance. A limited number of samples from a specific class can influence the learning process of the neural network. Different input configurations, such as reducing channels or applying linear transformations (e.g., PCA or IDT), can also impact the performance in recognizing individual classes. For instance, a linear transformation that accentuates anomalous behavior in a particular pathology, or the exclusion of leads that are clinically irrelevant for detecting a pathology, may significantly affect the model’s performance.

Based on these factors, weights were determined based on the inverse of the number of data points for each class $\left(n_{c}\right)$ and a λ parameter as:

$$w_{c}={\left(\frac{n_{c}}{\sum_{i}n_{i}}\right)}^{\lambda }$$ (6)

Equation 6 assigns greater weight to the minority classes and *vice versa*. The parameter λ plays a critical role in determining how effectively the model learns each class in an imbalanced dataset. When λ exceeds 1, the model exaggerates the difference in assigned weights, allowing it to focus more on instances from these minority classes during training. Conversely, when λ is less than 1, the model tends to distribute weights more evenly across all classes.

Given the two outlined factors—class imbalance and input transformations—we devised a strategy to search for the optimal value of λ that better weights each class for every designed input configuration, explained in detail in Supplementary Appendix SC.

### 3.4 Model evaluation

Each trained model underwent evaluation using various metrics to gain a comprehensive understanding of its behavior in different scenarios. Each metric assesses the model’s performance by measuring the degree of alignment between the predicted labels and the actual ones. The employed metrics are gathered in Table 1.

**TABLE 1:** Metrics and their equations.

Metric	Equation	Description
SN	$\text{SN}=\frac{\text{TP}}{\text{TP}+\text{FN}}$	Sensitivity (SN), also known as True Positive Rate (TPR) or Recall (Rec), measures the proportion of actual positives that are correctly identified by the model
SP	$\text{SP}=\frac{\text{TN}}{\text{TN}+\text{FP}}$	Specificity (SP) measures the proportion of actual negatives that are correctly identified by the model
Prec	$\text{Prec}=\frac{\text{TP}}{\text{TP}+\text{FP}}$	Precision (Prec) measures the proportion of positive identifications that are actually correct
AUPRC	$\text{AUPRC}=\int_{0}^{1}\text{Prec}\left(\text{Rec}\right)\;d\text{Rec}$	AUPRC is the area under the Precision-Recall curve
AUC	$\text{AUC}=\int_{0}^{1}\text{TPR}\left(\text{FPR}\right)\;d\text{FPR}$	AUC is the area under the Receiver Operating Characteristic (ROC) curve
F1 score	$F1=\frac{2\times \text{Prec}\times \text{SN}}{\text{Prec}+\text{SN}}$	F1 score is the harmonic mean of precision and recall

## 4 Results

The results highlighting the contributions of this work are organized as follows: first, we present the analysis of redundancy for each data subset. Subsequently, we detail their overall classification performance. Finally, we provide a breakdown by pathology. For additional results, refer to Supplementary Appendix SD.

### 4.1 Trainable parameters

As the number of channels decreases, the count of trainable model parameters diminishes, with a potential reduction of up to 27.13% when only one channel is used. Table 2 presents these findings. This parameter reduction is directly influenced by the architecture of the convolutional network, particularly in convolutional layer 6 (see Supplementary Appendix Figure S1).

**TABLE 2:** Macro average results in % for all metrics: Sensitivity (SN), Specificity (SP), Precision (Prec.), Area Under Precision-Recall Curve (AUPRC), Area Under the Curve (AUC), and F1 score, along with the redundancy metric (R) and the total number of parameters for each input model.

		Input	R	Params	SN	SP	Prec	AUPRC	AUC	F1
R Augm.		ECG 15	71.23	178,373 (7.40%)	**74.49**	**94.78**	70.48	73.88	*93.97*	71.32
Baseline		ECG 12	64.08	166,085 (0%)	68.74	94.53	**79.50**	**75.51**	**94.43**	*73.35*
R Red.	Lead Selection	ECG 8	27.46	149,701 (−9.86%)	*68.82*	*94.59*	74.83	74.83	93.60	73.09
		ECG 6	24.42	141,509 (−14.80%)	69.67	94.48	*78.00*	*75.20*	93.29	**73.38**
		ECG 3	16.46	129,221 (−22.20%)	60.89	93.46	72.22	68.22	91.54	65.40
		ECG 1	0	121,029 (−27.13%)	49.58	89.36	72.44	60.77	83.06	55.59
	Lead Transform	IDT	17.13	129,221 (−22.20%)	64.69	94.24	71.15	69.00	92.73	67.69
		PCA 8	17.33	149,701 (−9.86%)	64.55	93.87	75.75	71.74	92.73	69.15
		PCA 6	19.78	141,509 (−14.80%)	66.99	94.14	74.92	72.39	92.60	70.51
		PCA 3	9.31	129,221 (−22.20%)	65.98	93.93	71.59	70.49	92.31	68.29
		PCA 1	0	121,029 (−27.13%)	52.73	93.95	62.62	58.96	83.61	52.62

Bold and italic values refer to the highest and second-highest F1 score for each class, respectively.

### 4.2 Redundancy analysis

Among equivalent numbers of channels, ECG exhibits the highest redundancy, followed by VCG and PCA. Both lead reduction and lead transformation strategies effectively decrease the inherent redundancy in the input, from 64.43% in the original input to 16.46% with 3 channels, 17.13% with IDT, and 9.31% with PCA of three components. Table 2 illustrates how PCA results in the least redundancy for a given number of channels.

### 4.3 Overall performance Implications of reducing ECG channels

**FIGURE 6:**
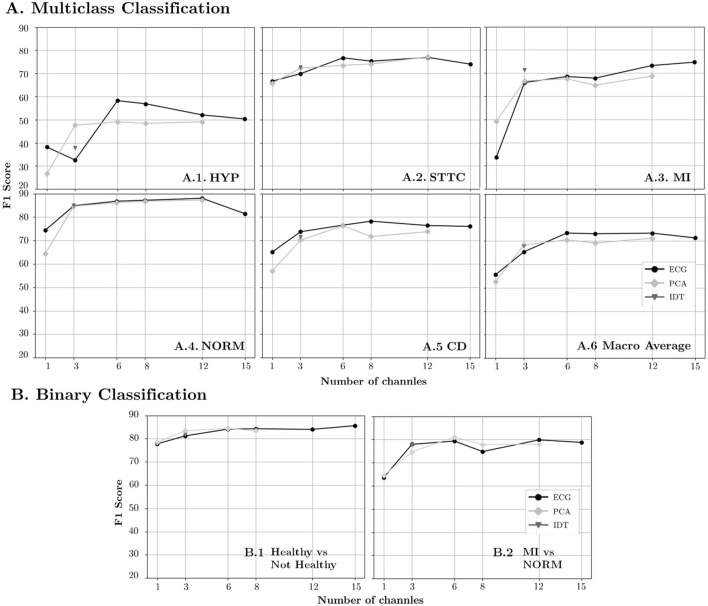
F1-Score results for diminishing number of channels in the original ECG representation and linear transformations (PCA and IDT) for **(A)** Multiclass Classification and **(B)** Binary Classification.

Both Table 2 and Figure 6 A.6 show that lead reduction results in a performance plateau from 12 to 6 leads in F1, with a variation of less than 0.29. However, there is a larger decrease in performance from 6 to 3 channels (7.98) and from 3 to 1 channel (18.06). The behavior with PCA is similar, though with only 3 components, both AUC and F1 are closer to saturation. Comparing the two redundancy reduction strategies, lead transformation with 3 channels (both VCG and PCA-3) outperformed the ECG. However, when extending to 6 channels, the ECG subset performed better than PCA-6. Furthermore, increasing redundancy to 15 channels was counterproductive, as all parameters worsened compared to the standard 12-lead ECG.

For the binary classification (see Figure 6B), the performance does not decrease by more than 2 points until 3 channels are reached. For 1 channel, ECG and PCA performance vary by less than 1 point in both classifications and are approximately 3 points below baseline in the Healthy vs. Not Healthy (Figure 6 B.1) scenario and 16 points in the MI vs. NORM case (Figure 6 B.2).

### 4.4 Pathology-specific performance analysis

The general trend across all pathologies is characterized by a plateau, a bend, and a decline in performance (see Table 3; Figures 6 A.1–A5). However, variations in behavior among the different pathologies warrant further analysis.

**TABLE 3:** F1 score results in % for each designed input.

		Input	R	HYP	STTC	MI	NORM	CD	H vs. NH	NORM vs. MI
R Augm		ECG 15	71.23	50.34	74.04	**74.72**	81.46	76.04	**85.62**	78.78
Baseline		ECG 12	64.08	52.08	**76.95**	*73.28*	**88.04**	76.41	84.03	**79.85**
R Red	Lead Selection	ECG 8	27.46	*56.88*	75.30	67.83	*87.19*	**78.26**	84.29	74.82
		ECG 6	24.42	**58.25**	*76.77*	68.52	86.79	*76.57*	84.13	*79.35*
		ECG 3	16.46	32.56	69.90	65.95	84.85	73.75	81.23	77.94
		ECG 1	0	38.16	66.67	33.62	74.40	65.10	77.78	63.70
	Lead Transform	IDT	17.13	37.84	72.72	71.31	84.99	71.59	81.87	77.56
		PCA 8	17.33	48.42	74.09	64.79	86.80	71.69	83.41	77.82
		PCA 6	19.78	49.06	73.52	67.52	86.14	76.30	*84.48*	81.12
		PCA 3	9.31	47.62	72.32	66.67	84.66	70.15	83.41	74.65
		PCA 1	0	26.62	65.79	49.16	64.46	57.07	78.51	64.54

Acronyms: R, Redundancy Score; HYP, Hypertrophy; STTC, ST-T Changes; MI, Myocardial Infarction; NORM, Normal; CD, Conduction Disturbance; H, Healthy; NH, Not Healthy. Bold and italic values refer to the highest and second-highest F1 score for each class, respectively.

PCA shows a pronounced increase in performance from 1 to 3 components, followed by a slight progression with fluctuations as the number of components increases. The performance of PCA-3 was similar to the ECG-3 subset, except for the HYP group, where PCA-3 outperformed ECG-3. The peak performance in the HYP group was achieved with the ECG-6 subset, which exceeded the baseline performance with 12 leads, reaching an F1 score of 58.25 (the lowest among all pathologies).

In the case of STTC, saturation was reached with ECG-6, achieving an F1 score of 76.77, higher than that of ECG-8. However, with only 3 components, both VCG transformations and PCA-3 outperformed ECG-3. Conversely, in the case of MI, greater redundancy led to optimal performance, with the input of 15 channels yielding the highest results with an F1 score of 74.72. For CD, the peak performance was achieved with ECG-8, using the full information, resulting in an F1 score of 78.26. Noticeably, adding more redundancy with 12 or 15 leads resulted in inferior outcomes.

Regarding normal ECGs, overall performance across all input types is superior to other pathologies. Reducing redundancy does not result in decreases greater than 3.4 points in F1 across all three channels (either ECG, VCG, or PCA).

## 5 Discussion

### 5.1 Redundancy management in ECG data

The redundancy in the 12-dimensional ECG is notably high, comprising 64.08% of the total information. When incorporating the VCG derived from linear transformations of the ECG, all new information becomes redundant, resulting in a total redundancy of 71.23% in the 15-lead ECG. The designed strategies effectively reduced redundancy by both selecting and transforming leads.

When considering inputs with three channels (ECG-3, IDT, and PCA-3), PCA demonstrates the least redundancy for two key reasons. Firstly, unlike IDT and the 3-lead ECG, PCA channels are perfectly orthogonal. Secondly, PCA captures more independent variables by representing variability along each of the three spatial axes. These findings are captured in Figure 6 and Table 2.

Once three orthogonal channels corresponding to spatial coordinates are obtained in PCA, adding three more channels, while they may provide explained variance, also introduces redundancy. However, when increasing from 6 to 8 components, the new channels have low amplitude, providing minimal information but also redundancy. Consequently, the proportion of total redundancy decreases. Finally, in PCA-12, the information is identical to that of PCA-8, and so is the redundancy, as the added channels are null, resulting in almost identical redundancy.

### 5.2 Global trends in model performance

In CNNs, the primary objective is to extract meaningful features while minimizing noise and irrelevant information. Redundant features fail to provide additional valuable insights for the learning process. By reducing redundancy, the learning process is streamlined, allowing the model to concentrate on the most informative features. This focus can lead to improved performance, as the network is not burdened with the task of filtering out or compensating for redundant data.

Additionally, efficiently reducing redundancy allows for a reduction in the number of model parameters to be trained without sacrificing information, thereby improving both the training and prediction processes. Such simplification is advantageous as it reduces computational complexity and enhances both training and inference efficiency. Consequently, the model becomes more focused and efficient in its learning process.

In the macro analysis, this effect is observed as the performance remains largely unaffected until the number of channels is reduced to between 6 and 3, representing a reduction in input size by half and a quarter, respectively (see Figure 6 A.6; Table 2).

This phenomenon arises from the cardiac dipole’s three-dimensional nature; thus, with just three channels, it is possible to capture all the necessary information with minimal redundancy. That said, the three sets of 3-channel inputs used in this study may still lose information for various reasons. In the case of lead selection, while efforts have been made to maximize orthogonality among the chosen leads, perfect orthogonality is not achievable, leading to distortion of the cardiac dipole. Similarly, in the case of PCA and VCG transformations, a single transformation is applied to all records without accounting for slight variations in the angles between leads during electrode placement, resulting in further distortions (see effect on Figure 6 A.6; Table 2).

Therefore, the optimal performance is achieved with 6 channels (Figure 6 A.6; Table 2), as the potential information distortions that occur with 3 channels are mitigated by redundancy without being significantly affected by excessive redundancy, which could lead to overfitting.

Although it is not possible to perform a direct comparison with the work of Lai et al. (2021), since they used a different database, deep learning architecture and pathologies, the results obtained are consistent with the conclusions of their study. Both their research and ours indicate that the use of three or fewer channels causes a notable decrease in the results.

Similar to how the VCG condenses all spatial information into just three projections, the ECG distributes the same information across 12 leads, thereby introducing inherent redundancy. The three spatial coordinates derived from the Frank leads are sufficient to capture all relevant features of the cardiac dipole without compromising any clinical information. When selecting leads based on reduced redundancy, there is no prior certainty that clinically relevant information from pathologies has not been discarded. However, the neural network results indicate that predictive performance remains consistent even when certain leads are excluded. This suggests that the criteria used to discard leads effectively eliminate redundant information while preserving the essential clinical features for all pathologies.

### 5.3 Do pathologies respond differently to redundancy reduction? Disease-specific analysis

The impact of information distortion varies across different pathologies, with some being more sensitive than others. In the case of MI, for example, including more leads proves more beneficial for detecting the pathology than the potential overfitting it may cause (Figure 6 A.3). One reason could be that the specific leads where the morphological changes of the ECG are reflected strongly depend on the location of the infarcted area, as described in (Hwang and Levis, 2014). Therefore, the removal of leads containing information about the location of the infarction may prove counterproductive in detecting it by the neural network. In contrast, for STTC, performance remains unaffected, with the reduction in redundancy compensating for possible information distortion (Figure 6 A.2). For HYP and CD, reducing redundancy has been beneficial, improving results with 6 and 8 channels, respectively (Figures 6 A.1, 6.A.5). Interestingly, for the NORM class, remarkable outcomes can be achieved with only 3 input leads (Figure 6 A.4). This suggests that a reduced subset could be adopted at an early stage for screening and fast discrimination of normal vs. abnormal ECGs, whereas a more complete input could be employed once an abnormal ECG has been detected.

Comparing redundancy reduction techniques, lead selection generally outperforms transformations across most numbers of channels and pathologies (see Table 3). Nonetheless, lead transformation yields better results in all pathologies except for CD when utilizing three channels to represent spatial coordinates (see Table 3). This implies that IDT and PCA better maintain orthogonality between channels and preserve information compared to lead selection. This finding holds particular significance in applications with a limited number of leads, such as in Holter devices or wearables, where maximizing orthogonality among recorded leads while reducing redundancy can be of interest to both clinicians and machine learning models.

Focusing on PCA transformation, we observe that with 3 orthogonal channels, the majority of the variance (93.50%) is explained. Notably, adding non-dipolar components does not significantly increase the explained variance. The results support this observation, as adding more than 3 components hardly changes the outcomes (Table 3). Specifically, when transitioning from 8 to 12 components, only 8 are independent leads, and the remaining 4 correspond to channels with practically insignificant amplitude. Therefore, the differences between the 8 and 12-channel PCA models may stem from the random initialization of parameters rather than the information within the input.

Since binary classification is a simpler task, the overall performance in both cases is slightly superior to that of multiclass classification (Figure 6). However, the trend with respect to redundancy is aligned with the results obtained in multiclass classification. This may be due to the fact that in both scenarios (multiclass classification and binary classification) the architecture of the feature extraction model is identical and the only thing that changes is the final class discrimination layer. Since redundancy affects the feature extraction part, it is consistent that it affects binary classification and multiclass classification in a similar way. In the case of healthy vs. unhealthy (Figure 6 B.1) the performance is very similar to the NORM case in multiclass classification, since the latter shows the NORM results against the rest just like the binary classification. The only difference is that while the former model has been trained on a multiclass classification task by training the model parameters for learning 5 classes, the latter has only focused on discriminating two. As for the MI vs. NORM (Figure 6 B.2) binary classification, the results tend to fluctuate more compared to the Healthy vs. Unhealthy classification. This variability can be attributed to two facts: the small number of samples for training and the smaller number of samples in the pathological class within the test set (256 samples), where a few errors can significantly affect the overall performance. However, if the performance of multiclass classification is compared with binary classification, a correspondence in trend can be observed, where 8-channel performance is lower than 6- and 12-channel performance in both PCA and ECG lead selection.

### 5.4 Limitations and future directions

In this study, we focused solely on the redundancy present in the input, which may be a limitation since preserving information could be also of interest. The next step would be to assess how much information from the original set of 12 leads is retained in each input studied to more clearly determine how input reduction influences the neural network.

An additional aspect that could be explored is the quantification of the similarity of the beats in time. In a 10-s recording, beats are cyclical and with a high degree of similarity mainly in healthy patients or even in certain pathologies. Evaluating the reduction of this temporal repetitiveness and condensing this information in a reduced number of samples could be an interesting object of study in the realm of a CNN.

While a 2D CNN model captures both spatial and temporal relationships, reducing input dimensions can increase model complexity and the risk of overfitting. Relying on a single model in the study may limit the generalizability of how redundancy affects CNNs. Therefore, future research should compare this model with one that treats each channel independently, eliminating dependency on input dimensionality. This comparison would allow the identification of the optimal modelling strategy by evaluating the effectiveness of capturing spatial and temporal relationships versus treating leads independently.

Finally, the study used a single dataset containing five cardiac conditions, focusing primarily on evaluating how redundancy affects CNN learning. Future research should explore additional datasets with the same or different conditions to validate the results and provide a more comprehensive evaluation. On the other hand, it would be clinically interesting to analyze the information of other clinical variables to maximize the results and link them with other clinical situations. The result obtained could be clinically relevant in different cardiovascular settings like coronary artery disease and cardiac arrhythmias.

## 6 Conclusion

In this study, we introduced a novel metric to quantify the redundancy within a set of channels. The developed metric has demonstrated two main functions. On the one hand, it provides an objective criterion to reduce the volume of data and the complexity of models in CNNs; on the other hand, it facilitates the selection of leads or even the development of new electrode configurations, especially in environments with a limited number of leads, as is the case with Holters or wearable devices. Our findings reveal that redundancy reduction does not exert uniform effects across all pathologies. Instead, it offers advantages in both performance enhancement and computational complexity reduction through redundancy reduction. Using 6 leads, we achieved nearly identical results compared to using 12 leads; when only 3 leads are used, transformations outperform the non-transformed leads. Additionally, employing 3 leads allows for efficient discrimination of normal ECGs, which is both faster and more cost-effective. These insights are particularly valuable for applications requiring fewer channels, such as enhancing efficiency in deep learning models or utilizing redundancy as a criterion for lead selection in Holter monitors or wearables.

## Data Availability

The original contributions presented in the study are included in the article/Supplementary Material, further inquiries can be directed to the corresponding author.
