# Supplementary material for: The art of selecting the ECG input in neural networks to classify heart diseases: a dual focus on maximizing information and reducing redundancy
Source: Front Physiol. 2024 Oct 7;15:1452829. doi: 10.3389/fphys.2024.1452829 (PMC11491564; doi:10.3389/fphys.2024.1452829)
Supplement: Supplementary file 1 [file DataSheet1.pdf]

# Supplementary Material

## APPENDIX A. INVERSE DOWER TRANSFORM

The VCG can be estimated from the Inverse Dower Transform (IDT) (Equation S1). Essentially, this method comprises a linear combination that projects the signals recorded by the standard 12-lead ECG onto the spatial coordinates XYZ:

$$\mathbf{vcg}(t) = \mathbf{IDT} \cdot \mathbf{ecg}(t), \tag{S1}$$

where $\mathbf{vcg}(t)$ is a 3-component vector containing the spatial leads XYZ, $\mathbf{ecg}(t)$ is an 8-component vector containing the 8 independent leads of the ECG (notice that from the 6 limb leads only two of them are independent and the rest of them can be computed from a linear combination, for example I and II) and $\mathbf{IDT}$ is a $3 \times 8$ matrix Table S1 representing the Inverse Dower Transform with the weights that each of the 8 independent ECG leads have on the XYZ components. Since the direct contribution of lead III and the augmented leads is disregarded in the IDT transformation, the second section of the ECG printout, containing the augmented leads exclusively, has absolutely no influence on the reconstructed $\mathbf{vcg}(t)$. Table S1 displays the coefficients of the IDT matrix, where it is worth mentioning the significant weight of lead II, particularly in generating the Y-coordinate.

**Table S1.** Transformation matrix coefficients (**IDT**) for Inverse Dower Transform.

| | I | II | V1 | V2 | V3 | V4 | V5 | V6 |
|---|---|---|---|---|---|---|---|---|
| X | 0.156 | -0.010 | -0.172 | -0.074 | 0.122 | 0.231 | 0.239 | 0.194 |
| Y | -0.227 | 0.887 | 0.057 | -0.019 | -0.106 | -0.022 | 0.041 | 0.048 |
| Z | 0.022 | 0.102 | -0.229 | -0.310 | -0.246 | -0.063 | 0.055 | 0.108 |

## APPENDIX B. NETWORK ARCHITECTURE

The architecture presented in (Anand et al., 2022), chosen for this study, is depicted in Figure S1.

## APPENDIX C. IMPLEMENTATION DETAILS

The table provided (Table S2) lists the hyperparameters utilized in each model. While the loss function and optimizer were selected according to findings in (Anand et al., 2022), we conducted a thorough search for learning rates and lambda parameters tailored to each input, as elaborated below.

### *C.1 Learning Rate search*

For the selection of the learning rate, an empirical search was conducted based on the results of the AUC on the validation set. To do this, a search range from $5 \times 10^{-6}$ to $5 \times 10^{-1}$ was explored.

### *C.2 $\lambda$ Parameter optimization*

The methodology to optimize $\lambda$ entails a systematic exploration of a predefined range of $\lambda$ values, typically ranging from 0 to 2 in increments of 0.25, while evaluating the model's performance on a validation dataset at each value. Initially, the exploration commences with a fixed step size, such as 0.25,

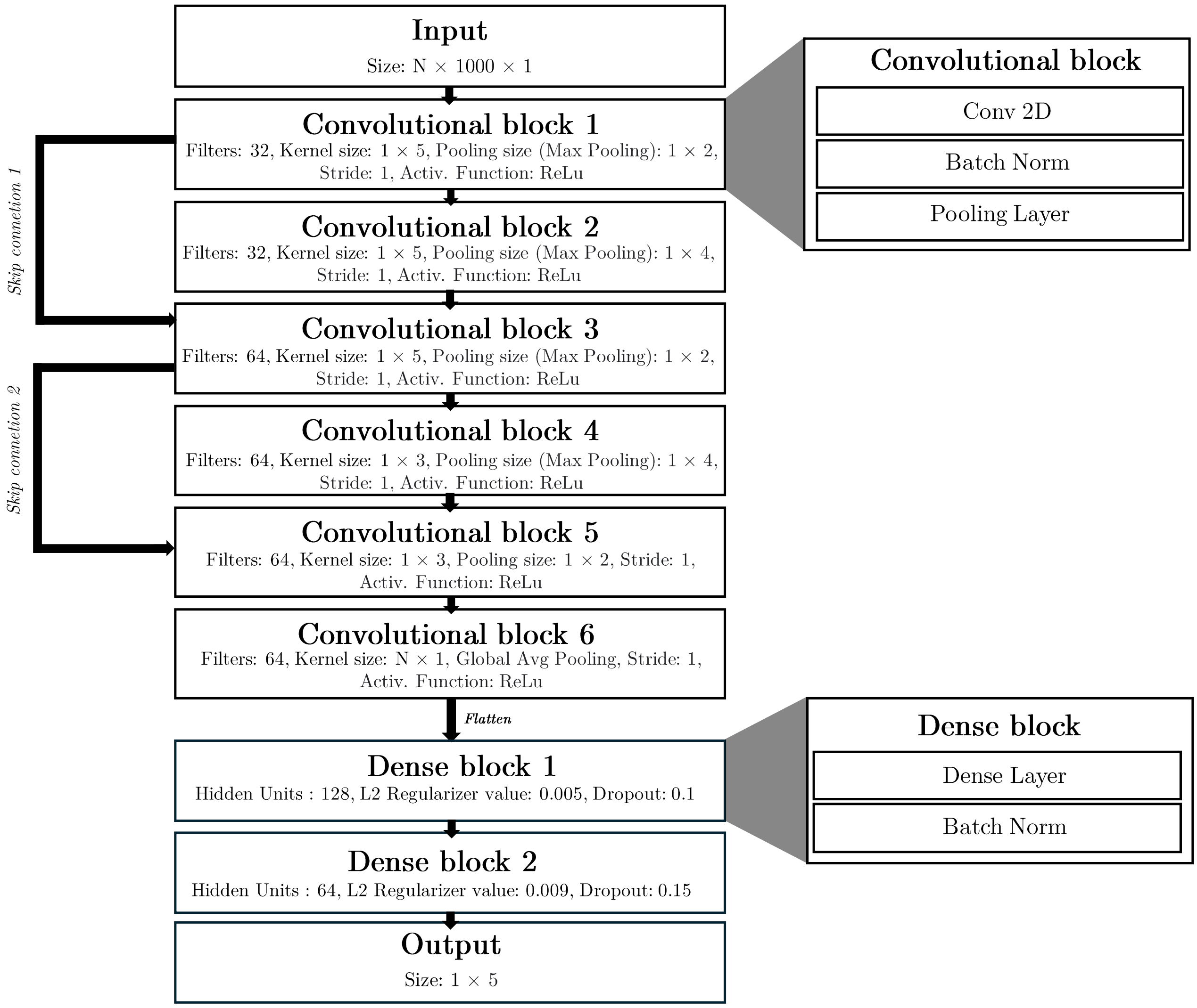


**Figure S1.** 2D-CNN backbone. $N$ denotes the number of input channels (i.e. $N = 12$ in standard ECG or $N = 3$ in VCG).

spanning the entire range. Should a notable performance enhancement be observed at a specific $\lambda$ value, the step size is halved. Subsequently, the search extends to neighboring $\lambda$ values on both sides of the improved point. If performance continues to exhibit improvement, the step size is further halved, and the search scope is adjusted accordingly. This iterative process persists until there is no substantial enhancement in both the mean and standard deviation, typically by 0.05.

To determine the optimal $\lambda$ factor for each model and address the imbalance effect, we computed the mean and standard deviation of the AUC for all models in the validation set. The selected $\lambda$ value is the one that exhibits the highest mean and lowest standard deviation. This choice indicates that this $\lambda$ parameter yields the best results for all classes in a relatively balanced manner.

**Table S2.** Hyperparameters used for training each model.

| | Loss Function | Optimizer | Learning rate | $\lambda$ |
|---|---|---|---|---|
| ECG 15 | BCE | Adam | 0.0005 | 0.3750 |
| ECG 12 | BCE | Adam | 0.0005 | 0.3750 |
| ECG 8 | BCE | Adam | 0.00045 | 0.4125 |
| ECG 6 | BCE | Adam | 0.0003 | 0.4750 |
| ECG 3 | BCE | Adam | 0.0001 | 0.5000 |
| ECG 1 | BCE | Adam | 0.00005 | 0.6250 |
| IDT | BCE | Adam | 0.0001 | 0.5000 |
| PCA 12 | BCE | Adam | 0.0005 | 0.4000 |
| PCA 8 | BCE | Adam | 0.00045 | 0.4500 |
| PCA 6 | BCE | Adam | 0.0003 | 0.4825 |
| PCA 3 | BCE | Adam | 0.0001 | 0.5625 |
| PCA 1 | BCE | Adam | 0.00005 | 0.7000 |

To demonstrate how the $\lambda$ value influences each input, we present various examples in Figure S2 using the same type of input, such as ECG, but with different numbers of channels (12 and 3). Additionally, we compare inputs with the same number of channels, such as 3, but with different input types, including ECG, PCA, and IDT. For the 12-lead ECG input, the optimal $\lambda$ value is approximately 0.375. However, for inputs with 3 channels, the $\lambda$ value increases to around 0.5 in all cases.

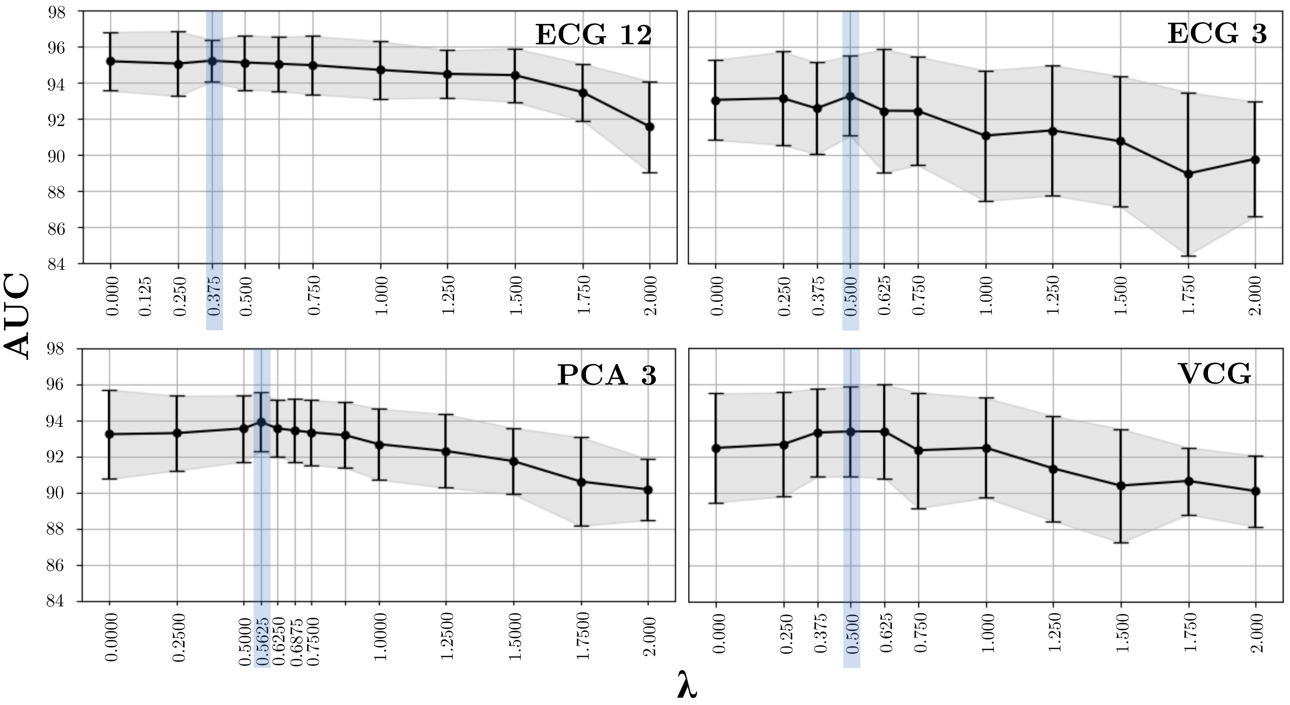


**Figure S2.** AUC performance depending on $\lambda$ value in varying number of channels (12 and 3) and information representation (ECG, PCA and VCG).

## REFERENCES

Anand, A., Kadian, T., Shetty, M. K., and Gupta, A. (2022). Explainable AI decision model for ECG data of cardiac disorders. *Biomedical Signal Processing and Control* 75, 103584
